# 1-Dodec­yloxy-4-nitro­benzene

**DOI:** 10.1107/S1600536809045966

**Published:** 2009-11-07

**Authors:** Xi-Gui Yue

**Affiliations:** a Affiliation: Alan G. MacDiarmid Institute, Jilin University, Changchun 130012, People’s Republic of China

## Abstract

The asymmetric unit of the title compound, C_18_H_29_NO_3_, contains two independent mol­ecules. The benzene ring and the mean plane of the alkyl unit form dihedral angles of 83.69 (12) and 77.14 (11)° in the two mol­ecules. In the crystal structure, weak C—H⋯O hydrogen bonds link mol­ecules into double-layer ribbons extending in [110].

## Related literature

For the structure of a related nitro­benzene derivative, see Yue (2009 ▶).
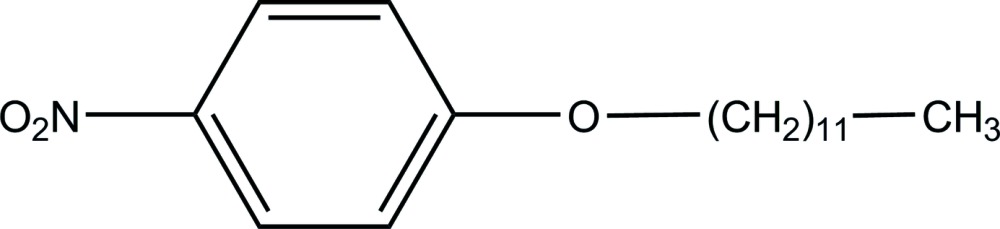


## Experimental

### 

#### Crystal data

C_18_H_29_NO_3_
*M* *_r_* = 307.42
Triclinic, 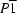

*a* = 5.615 (3) Å
*b* = 16.064 (7) Å
*c* = 21.390 (12) Å
α = 72.190 (15)°
β = 87.290 (18)°
γ = 80.240 (16)°
*V* = 1810.1 (15) Å^3^
*Z* = 4
Mo *K*α radiation
μ = 0.08 mm^−1^
*T* = 291 K
0.23 × 0.23 × 0.21 mm

#### Data collection

Rigaku R-AXIS RAPID diffractometer
Absorption correction: multi-scan (*ABSCOR*; Higashi, 1995 ▶) *T*_min_ = 0.983, *T*_max_ = 0.984
13636 measured reflections
6158 independent reflections
2548 reflections with *I* > 2σ(*I*)
*R*_int_ = 0.064

#### Refinement

*R*[*F* ^2^ > 2σ(*F* ^2^)] = 0.068
*wR*(*F* ^2^) = 0.180
*S* = 0.99
6158 reflections
399 parameters
H-atom parameters constrained
Δρ_max_ = 0.15 e Å^−3^
Δρ_min_ = −0.16 e Å^−3^

### 

Data collection: *RAPID-AUTO* (Rigaku, 1998 ▶); cell refinement: *RAPID-AUTO*; data reduction: *CrystalStructure* (Rigaku/MSC, 2002 ▶); program(s) used to solve structure: *SHELXS97* (Sheldrick, 2008 ▶); program(s) used to refine structure: *SHELXL97* (Sheldrick, 2008 ▶); molecular graphics: *PLATON* (Spek, 2009 ▶); software used to prepare material for publication: *SHELXL97*.

## Supplementary Material

Crystal structure: contains datablocks global, I. DOI: 10.1107/S1600536809045966/cv2628sup1.cif

Structure factors: contains datablocks I. DOI: 10.1107/S1600536809045966/cv2628Isup2.hkl

Additional supplementary materials:  crystallographic information; 3D view; checkCIF report

## Figures and Tables

**Table 1:** Hydrogen-bond geometry (Å, °)

*D*—H⋯*A*	*D*—H	H⋯*A*	*D*⋯*A*	*D*—H⋯*A*
C3—H3⋯O4^i^	0.93	2.69	3.329 (4)	127
C6—H6⋯O2^ii^	0.93	2.60	3.375 (4)	141
C20—H20⋯O5^iii^	0.93	2.52	3.372 (5)	152

Symmetry codes: (i) 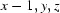; (ii) 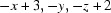; (iii) 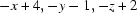.

**Fig. 1.**
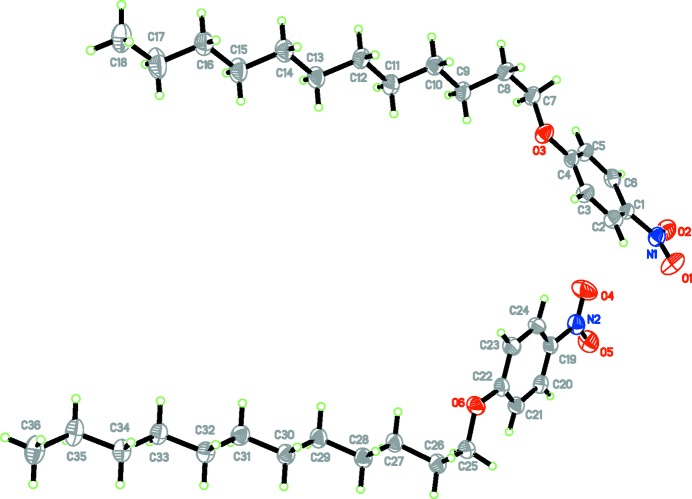
Two independent molecules of the title compound showing the atom numbering and 30% probability displacement ellipsoids.

## supplementary crystallographic information

### Comment

Nitrobenzene and its derivatives are of great interest for their various applications. Recently, we reported the crystal structure of 1-decyloxy-4-nitrobenzene (Yue, 2009). As an extension of our work on the structure characterizations of nitrobenzene derivatives, we report herein the crystal structure of the title compound.

The title compound, as shown in Fig. 1, comprises two independent molecules in the asymmetric unit. Two benzene rings of the two molecules form a dihedral angle of 48.12 (13) °. Weak C—H···O hydrogen bonds (Table 1) link molecules into ribbons extended in direction [110].

### Experimental

4-Nitrophenol (0.14 g, 1 mmol) and dodecyl iodide (0.30 g, 1 mmol) were dissovled in 15 ml of acetone. The sodium hydroxide solution (10 ml, 8%) was added into the above solution. The resultant mixture was heated for 2 h under refluxing and then the solution was cooled to room temperaure in an ice bath with stirring. The colourless crystals suitable for single-crystal analysis were obtained by recrystallization from 95% ethanol.

### Refinement

H atoms were placed in calculated positions (C—H 0.93-0.97 Å) and were included in the refinement in the riding model with *U*_iso_(H) = 1.2 or 1.5 *U*_eq_(C).

### Crystal data

**Table:**

C_18_H_29_NO_3_	*Z* = 4
*M**_r_* = 307.42	*F*(000) = 672
Triclinic, *P*1	*D*_x_ = 1.128 Mg m^−^^3^
Hall symbol: -P 1	Mo *K*α radiation, λ = 0.71073 Å
*a* = 5.615 (3) Å	Cell parameters from 8462 reflections
*b* = 16.064 (7) Å	θ = 3.0–25.0°
*c* = 21.390 (12) Å	µ = 0.08 mm^−^^1^
α = 72.190 (15)°	*T* = 291 K
β = 87.290 (18)°	Block, colourless
γ = 80.240 (16)°	0.23 × 0.23 × 0.21 mm
*V* = 1810.1 (15) Å^3^	

### Data collection

**Table:**

Rigaku R-AXIS RAPID diffractometer	6158 independent reflections
Radiation source: fine-focus sealed tube	2548 reflections with *I* > 2σ(*I*)
graphite	*R*_int_ = 0.064
ω scans	θ_max_ = 25.0°, θ_min_ = 3.0°
Absorption correction: multi-scan (*ABSCOR*; Higashi, 1995)	*h* = −6→5
*T*_min_ = 0.983, *T*_max_ = 0.984	*k* = −18→19
13636 measured reflections	*l* = −25→25

### Refinement

**Table:**

Refinement on *F*^2^	Primary atom site location: structure-invariant direct methods
Least-squares matrix: full	Secondary atom site location: difference Fourier map
*R*[*F*^2^ > 2σ(*F*^2^)] = 0.068	Hydrogen site location: inferred from neighbouring sites
*wR*(*F*^2^) = 0.180	H-atom parameters constrained
*S* = 0.99	*w* = 1/[σ^2^(*F*_o_^2^) + (0.0744*P*)^2^] where *P* = (*F*_o_^2^ + 2*F*_c_^2^)/3
6158 reflections	(Δ/σ)_max_ < 0.001
399 parameters	Δρ_max_ = 0.15 e Å^−^^3^
0 restraints	Δρ_min_ = −0.16 e Å^−^^3^

### Special details

**Table:**

Experimental. (See detailed section in the paper)
Geometry. All e.s.d.'s (except the e.s.d. in the dihedral angle between two l.s. planes) are estimated using the full covariance matrix. The cell e.s.d.'s are taken into account individually in the estimation of e.s.d.'s in distances, angles and torsion angles; correlations between e.s.d.'s in cell parameters are only used when they are defined by crystal symmetry. An approximate (isotropic) treatment of cell e.s.d.'s is used for estimating e.s.d.'s involving l.s. planes.
Refinement. Refinement of *F*^2^ against ALL reflections. The weighted *R*-factor *wR* and goodness of fit *S* are based on *F*^2^, conventional *R*-factors *R* are based on *F*, with *F* set to zero for negative *F*^2^. The threshold expression of *F*^2^ > σ(*F*^2^) is used only for calculating *R*-factors(gt) *etc*. and is not relevant to the choice of reflections for refinement. *R*-factors based on *F*^2^ are statistically about twice as large as those based on *F*, and *R*- factors based on ALL data will be even larger.

### Fractional atomic coordinates and isotropic or equivalent isotropic displacement parameters (Å^2^)

**Table:**

	*x*	*y*	*z*	*U*_iso_*/*U*_eq_	
C1	1.1633 (6)	−0.1114 (2)	0.98321 (16)	0.0464 (9)	
C2	0.9771 (7)	−0.1433 (2)	0.96353 (19)	0.0577 (11)	
H2	0.9392	−0.1982	0.9877	0.069*	
C3	0.8480 (7)	−0.0939 (2)	0.90832 (17)	0.0544 (10)	
H3	0.7243	−0.1159	0.8943	0.065*	
C4	0.9005 (6)	−0.0111 (2)	0.87311 (16)	0.0482 (9)	
C5	1.0903 (6)	0.0201 (2)	0.89214 (17)	0.0513 (10)	
H5	1.1290	0.0748	0.8678	0.062*	
C6	1.2222 (7)	−0.0301 (2)	0.94732 (17)	0.0548 (10)	
H6	1.3504	−0.0094	0.9605	0.066*	
C7	0.7796 (7)	0.1225 (2)	0.78507 (17)	0.0593 (11)	
H7A	0.9356	0.1240	0.7636	0.071*	
H7B	0.7679	0.1582	0.8147	0.071*	
C8	0.5811 (7)	0.1577 (2)	0.73523 (17)	0.0594 (11)	
H8A	0.5787	0.2209	0.7158	0.071*	
H8B	0.4283	0.1498	0.7575	0.071*	
C9	0.5996 (7)	0.1151 (2)	0.68065 (17)	0.0576 (11)	
H9A	0.6023	0.0519	0.6998	0.069*	
H9B	0.7511	0.1236	0.6578	0.069*	
C10	0.3935 (7)	0.1523 (2)	0.63141 (17)	0.0582 (10)	
H10A	0.2426	0.1427	0.6543	0.070*	
H10B	0.3886	0.2158	0.6132	0.070*	
C11	0.4119 (7)	0.1121 (2)	0.57567 (18)	0.0622 (11)	
H11A	0.5619	0.1225	0.5524	0.075*	
H11B	0.4190	0.0486	0.5939	0.075*	
C12	0.2058 (7)	0.1482 (2)	0.52735 (18)	0.0605 (11)	
H12A	0.1992	0.2118	0.5092	0.073*	
H12B	0.0561	0.1381	0.5508	0.073*	
C13	0.2195 (7)	0.1091 (2)	0.47143 (19)	0.0650 (11)	
H13A	0.3689	0.1194	0.4479	0.078*	
H13B	0.2270	0.0455	0.4896	0.078*	
C14	0.0136 (7)	0.1446 (3)	0.42319 (19)	0.0701 (12)	
H14A	0.0065	0.2081	0.4048	0.084*	
H14B	−0.1360	0.1344	0.4467	0.084*	
C15	0.0286 (8)	0.1047 (3)	0.3678 (2)	0.0761 (13)	
H15A	0.0399	0.0410	0.3863	0.091*	
H15B	0.1767	0.1160	0.3438	0.091*	
C16	−0.1797 (8)	0.1382 (3)	0.3197 (2)	0.0740 (13)	
H16A	−0.3277	0.1273	0.3439	0.089*	
H16B	−0.1902	0.2019	0.3011	0.089*	
C17	−0.1683 (9)	0.0991 (4)	0.2650 (2)	0.1038 (17)	
H17A	−0.1457	0.0351	0.2835	0.125*	
H17B	−0.0266	0.1138	0.2389	0.125*	
C18	−0.3825 (8)	0.1275 (3)	0.2203 (2)	0.0944 (16)	
H18A	−0.5184	0.1035	0.2434	0.142*	
H18B	−0.3471	0.1062	0.1830	0.142*	
H18C	−0.4200	0.1910	0.2058	0.142*	
C19	1.5593 (7)	−0.3938 (2)	0.92787 (16)	0.0513 (10)	
C20	1.6746 (7)	−0.4774 (2)	0.93077 (16)	0.0531 (10)	
H20	1.8218	−0.5002	0.9526	0.064*	
C21	1.5700 (7)	−0.5274 (2)	0.90099 (17)	0.0531 (10)	
H21	1.6470	−0.5839	0.9022	0.064*	
C22	1.3504 (7)	−0.4930 (2)	0.86934 (16)	0.0513 (10)	
C23	1.2366 (7)	−0.4080 (2)	0.86619 (18)	0.0584 (11)	
H23	1.0905	−0.3846	0.8438	0.070*	
C24	1.3406 (7)	−0.3587 (2)	0.89620 (17)	0.0561 (10)	
H24	1.2642	−0.3022	0.8951	0.067*	
C25	1.3257 (7)	−0.6272 (2)	0.84394 (18)	0.0613 (11)	
H25A	1.3344	−0.6628	0.8897	0.074*	
H25B	1.4870	−0.6319	0.8256	0.074*	
C26	1.1596 (7)	−0.6589 (2)	0.80708 (17)	0.0622 (11)	
H26A	0.9977	−0.6498	0.8244	0.075*	
H26B	1.2090	−0.7221	0.8150	0.075*	
C27	1.1516 (7)	−0.6140 (2)	0.73391 (17)	0.0569 (11)	
H27A	1.3134	−0.6227	0.7165	0.068*	
H27B	1.1005	−0.5508	0.7258	0.068*	
C28	0.9838 (7)	−0.6477 (2)	0.69766 (17)	0.0577 (11)	
H28A	1.0324	−0.7112	0.7073	0.069*	
H28B	0.8217	−0.6375	0.7147	0.069*	
C29	0.9752 (7)	−0.6064 (2)	0.62407 (17)	0.0572 (11)	
H29A	1.1368	−0.6165	0.6068	0.069*	
H29B	0.9256	−0.5428	0.6142	0.069*	
C30	0.8054 (7)	−0.6419 (2)	0.58938 (17)	0.0588 (11)	
H30A	0.8532	−0.7056	0.6001	0.071*	
H30B	0.6435	−0.6307	0.6062	0.071*	
C31	0.7980 (7)	−0.6027 (2)	0.51597 (18)	0.0603 (11)	
H31A	0.9597	−0.6138	0.4990	0.072*	
H31B	0.7495	−0.5391	0.5051	0.072*	
C32	0.6273 (7)	−0.6392 (2)	0.48198 (17)	0.0577 (11)	
H32A	0.6749	−0.7030	0.4935	0.069*	
H32B	0.4657	−0.6276	0.4988	0.069*	
C33	0.6188 (7)	−0.6017 (2)	0.40820 (17)	0.0606 (11)	
H33A	0.7798	−0.6141	0.3912	0.073*	
H33B	0.5733	−0.5378	0.3966	0.073*	
C34	0.4454 (7)	−0.6375 (2)	0.37487 (18)	0.0613 (11)	
H34A	0.4905	−0.7014	0.3868	0.074*	
H34B	0.2844	−0.6249	0.3918	0.074*	
C35	0.4363 (8)	−0.6012 (3)	0.30168 (19)	0.0780 (13)	
H35A	0.5972	−0.6137	0.2847	0.094*	
H35B	0.3903	−0.5374	0.2896	0.094*	
C36	0.2635 (8)	−0.6377 (3)	0.2690 (2)	0.0848 (14)	
H36A	0.1016	−0.6221	0.2830	0.127*	
H36B	0.3056	−0.7011	0.2810	0.127*	
H36C	0.2735	−0.6132	0.2222	0.127*	
N1	1.2958 (6)	−0.1617 (2)	1.04342 (15)	0.0629 (9)	
N2	1.6664 (7)	−0.3418 (3)	0.96029 (16)	0.0653 (10)	
O1	1.2231 (5)	−0.22878 (19)	1.07737 (14)	0.0902 (10)	
O2	1.4686 (5)	−0.13502 (17)	1.05870 (13)	0.0791 (9)	
O3	0.7542 (5)	0.03322 (15)	0.82063 (12)	0.0614 (7)	
O4	1.5594 (6)	−0.2675 (2)	0.95704 (17)	0.1001 (12)	
O5	1.8548 (6)	−0.37346 (19)	0.99037 (15)	0.0833 (10)	
O6	1.2305 (4)	−0.53613 (15)	0.83828 (12)	0.0608 (7)	

### Atomic displacement parameters (Å^2^)

**Table:**

	*U*^11^	*U*^22^	*U*^33^	*U*^12^	*U*^13^	*U*^23^
C1	0.049 (2)	0.048 (2)	0.040 (2)	−0.0047 (18)	−0.0020 (18)	−0.0123 (16)
C2	0.058 (3)	0.054 (2)	0.063 (3)	−0.018 (2)	0.002 (2)	−0.0157 (19)
C3	0.056 (3)	0.056 (2)	0.052 (2)	−0.016 (2)	−0.012 (2)	−0.0131 (18)
C4	0.054 (2)	0.054 (2)	0.039 (2)	−0.0096 (19)	−0.0050 (19)	−0.0157 (17)
C5	0.057 (3)	0.054 (2)	0.045 (2)	−0.021 (2)	−0.003 (2)	−0.0119 (17)
C6	0.052 (2)	0.065 (2)	0.054 (3)	−0.015 (2)	−0.001 (2)	−0.0240 (19)
C7	0.079 (3)	0.054 (2)	0.045 (2)	−0.017 (2)	−0.007 (2)	−0.0109 (17)
C8	0.071 (3)	0.056 (2)	0.047 (2)	−0.008 (2)	−0.011 (2)	−0.0102 (18)
C9	0.066 (3)	0.064 (2)	0.045 (2)	−0.012 (2)	−0.005 (2)	−0.0184 (18)
C10	0.057 (3)	0.066 (2)	0.051 (2)	−0.005 (2)	−0.005 (2)	−0.0178 (19)
C11	0.068 (3)	0.070 (2)	0.051 (2)	−0.011 (2)	−0.007 (2)	−0.0208 (19)
C12	0.058 (3)	0.075 (2)	0.047 (2)	−0.004 (2)	−0.006 (2)	−0.0195 (19)
C13	0.058 (3)	0.082 (3)	0.059 (3)	0.000 (2)	−0.012 (2)	−0.032 (2)
C14	0.069 (3)	0.086 (3)	0.060 (3)	−0.007 (2)	−0.009 (2)	−0.032 (2)
C15	0.075 (3)	0.098 (3)	0.062 (3)	−0.010 (3)	−0.015 (2)	−0.035 (2)
C16	0.080 (3)	0.086 (3)	0.060 (3)	−0.009 (2)	−0.009 (2)	−0.029 (2)
C17	0.092 (4)	0.157 (4)	0.083 (4)	−0.005 (3)	−0.019 (3)	−0.071 (3)
C18	0.102 (4)	0.119 (4)	0.073 (3)	−0.023 (3)	−0.021 (3)	−0.039 (3)
C19	0.063 (3)	0.055 (2)	0.042 (2)	−0.022 (2)	0.003 (2)	−0.0170 (17)
C20	0.052 (2)	0.066 (2)	0.042 (2)	−0.012 (2)	−0.0028 (18)	−0.0159 (18)
C21	0.053 (3)	0.056 (2)	0.051 (2)	−0.0061 (19)	−0.003 (2)	−0.0186 (18)
C22	0.056 (3)	0.060 (2)	0.043 (2)	−0.016 (2)	0.0008 (19)	−0.0193 (18)
C23	0.055 (3)	0.060 (2)	0.061 (3)	−0.004 (2)	−0.006 (2)	−0.0212 (19)
C24	0.064 (3)	0.049 (2)	0.059 (3)	−0.009 (2)	0.004 (2)	−0.0223 (18)
C25	0.077 (3)	0.056 (2)	0.053 (3)	−0.007 (2)	−0.009 (2)	−0.0200 (18)
C26	0.078 (3)	0.063 (2)	0.053 (3)	−0.021 (2)	−0.003 (2)	−0.0233 (19)
C27	0.066 (3)	0.061 (2)	0.050 (2)	−0.019 (2)	−0.003 (2)	−0.0219 (18)
C28	0.061 (3)	0.062 (2)	0.055 (3)	−0.018 (2)	0.001 (2)	−0.0221 (18)
C29	0.063 (3)	0.065 (2)	0.049 (2)	−0.018 (2)	−0.004 (2)	−0.0201 (19)
C30	0.066 (3)	0.062 (2)	0.054 (3)	−0.020 (2)	−0.006 (2)	−0.0199 (18)
C31	0.060 (3)	0.065 (2)	0.056 (3)	−0.013 (2)	−0.009 (2)	−0.0158 (19)
C32	0.056 (3)	0.064 (2)	0.054 (3)	−0.012 (2)	−0.002 (2)	−0.0179 (18)
C33	0.065 (3)	0.071 (2)	0.046 (2)	−0.015 (2)	−0.005 (2)	−0.0155 (19)
C34	0.061 (3)	0.074 (2)	0.052 (3)	−0.013 (2)	−0.003 (2)	−0.0228 (19)
C35	0.076 (3)	0.110 (3)	0.051 (3)	−0.025 (3)	−0.008 (2)	−0.021 (2)
C36	0.074 (3)	0.115 (3)	0.067 (3)	−0.015 (3)	−0.019 (3)	−0.028 (3)
N1	0.066 (2)	0.070 (2)	0.051 (2)	−0.0071 (19)	−0.0105 (18)	−0.0163 (17)
N2	0.072 (3)	0.074 (2)	0.060 (2)	−0.031 (2)	0.004 (2)	−0.0266 (19)
O1	0.093 (2)	0.0818 (19)	0.077 (2)	−0.0241 (18)	−0.0156 (18)	0.0116 (16)
O2	0.080 (2)	0.0864 (19)	0.069 (2)	−0.0167 (17)	−0.0267 (17)	−0.0151 (15)
O3	0.0731 (19)	0.0588 (15)	0.0521 (16)	−0.0209 (14)	−0.0134 (14)	−0.0092 (12)
O4	0.107 (3)	0.076 (2)	0.136 (3)	−0.015 (2)	−0.021 (2)	−0.056 (2)
O5	0.077 (2)	0.098 (2)	0.090 (2)	−0.0213 (18)	−0.0191 (19)	−0.0434 (17)
O6	0.0667 (18)	0.0597 (15)	0.0628 (17)	−0.0066 (14)	−0.0164 (14)	−0.0282 (12)

### Geometric parameters (Å, °)

**Table:**

C1—C2	1.373 (4)	C19—N2	1.451 (4)
C1—C6	1.384 (4)	C20—C21	1.380 (4)
C1—N1	1.454 (4)	C20—H20	0.9300
C2—C3	1.365 (4)	C21—C22	1.379 (5)
C2—H2	0.9300	C21—H21	0.9300
C3—C4	1.387 (4)	C22—O6	1.362 (4)
C3—H3	0.9300	C22—C23	1.389 (5)
C4—O3	1.357 (4)	C23—C24	1.373 (4)
C4—C5	1.378 (4)	C23—H23	0.9300
C5—C6	1.374 (4)	C24—H24	0.9300
C5—H5	0.9300	C25—O6	1.440 (4)
C6—H6	0.9300	C25—C26	1.492 (4)
C7—O3	1.432 (3)	C25—H25A	0.9700
C7—C8	1.492 (4)	C25—H25B	0.9700
C7—H7A	0.9700	C26—C27	1.509 (5)
C7—H7B	0.9700	C26—H26A	0.9700
C8—C9	1.515 (5)	C26—H26B	0.9700
C8—H8A	0.9700	C27—C28	1.510 (4)
C8—H8B	0.9700	C27—H27A	0.9700
C9—C10	1.517 (4)	C27—H27B	0.9700
C9—H9A	0.9700	C28—C29	1.509 (4)
C9—H9B	0.9700	C28—H28A	0.9700
C10—C11	1.512 (5)	C28—H28B	0.9700
C10—H10A	0.9700	C29—C30	1.515 (4)
C10—H10B	0.9700	C29—H29A	0.9700
C11—C12	1.504 (5)	C29—H29B	0.9700
C11—H11A	0.9700	C30—C31	1.502 (5)
C11—H11B	0.9700	C30—H30A	0.9700
C12—C13	1.506 (5)	C30—H30B	0.9700
C12—H12A	0.9700	C31—C32	1.519 (4)
C12—H12B	0.9700	C31—H31A	0.9700
C13—C14	1.502 (5)	C31—H31B	0.9700
C13—H13A	0.9700	C32—C33	1.507 (5)
C13—H13B	0.9700	C32—H32A	0.9700
C14—C15	1.503 (5)	C32—H32B	0.9700
C14—H14A	0.9700	C33—C34	1.514 (4)
C14—H14B	0.9700	C33—H33A	0.9700
C15—C16	1.508 (5)	C33—H33B	0.9700
C15—H15A	0.9700	C34—C35	1.494 (5)
C15—H15B	0.9700	C34—H34A	0.9700
C16—C17	1.483 (6)	C34—H34B	0.9700
C16—H16A	0.9700	C35—C36	1.510 (5)
C16—H16B	0.9700	C35—H35A	0.9700
C17—C18	1.491 (5)	C35—H35B	0.9700
C17—H17A	0.9700	C36—H36A	0.9600
C17—H17B	0.9700	C36—H36B	0.9600
C18—H18A	0.9600	C36—H36C	0.9600
C18—H18B	0.9600	N1—O2	1.220 (3)
C18—H18C	0.9600	N1—O1	1.224 (3)
C19—C20	1.373 (5)	N2—O5	1.214 (4)
C19—C24	1.377 (5)	N2—O4	1.225 (4)
			
C2—C1—C6	120.6 (3)	C19—C20—H20	120.3
C2—C1—N1	120.2 (3)	C21—C20—H20	120.3
C6—C1—N1	119.2 (3)	C22—C21—C20	119.6 (4)
C3—C2—C1	119.6 (3)	C22—C21—H21	120.2
C3—C2—H2	120.2	C20—C21—H21	120.2
C1—C2—H2	120.2	O6—C22—C21	124.7 (3)
C2—C3—C4	120.2 (3)	O6—C22—C23	114.8 (3)
C2—C3—H3	119.9	C21—C22—C23	120.5 (3)
C4—C3—H3	119.9	C24—C23—C22	119.7 (4)
O3—C4—C5	125.0 (3)	C24—C23—H23	120.1
O3—C4—C3	114.9 (3)	C22—C23—H23	120.1
C5—C4—C3	120.1 (3)	C23—C24—C19	119.3 (4)
C6—C5—C4	119.7 (3)	C23—C24—H24	120.4
C6—C5—H5	120.2	C19—C24—H24	120.4
C4—C5—H5	120.2	O6—C25—C26	107.4 (3)
C5—C6—C1	119.7 (3)	O6—C25—H25A	110.2
C5—C6—H6	120.1	C26—C25—H25A	110.2
C1—C6—H6	120.1	O6—C25—H25B	110.2
O3—C7—C8	107.4 (3)	C26—C25—H25B	110.2
O3—C7—H7A	110.2	H25A—C25—H25B	108.5
C8—C7—H7A	110.2	C25—C26—C27	114.7 (3)
O3—C7—H7B	110.2	C25—C26—H26A	108.6
C8—C7—H7B	110.2	C27—C26—H26A	108.6
H7A—C7—H7B	108.5	C25—C26—H26B	108.6
C7—C8—C9	115.1 (3)	C27—C26—H26B	108.6
C7—C8—H8A	108.5	H26A—C26—H26B	107.6
C9—C8—H8A	108.5	C26—C27—C28	113.6 (3)
C7—C8—H8B	108.5	C26—C27—H27A	108.8
C9—C8—H8B	108.5	C28—C27—H27A	108.8
H8A—C8—H8B	107.5	C26—C27—H27B	108.8
C8—C9—C10	113.3 (3)	C28—C27—H27B	108.8
C8—C9—H9A	108.9	H27A—C27—H27B	107.7
C10—C9—H9A	108.9	C29—C28—C27	115.5 (3)
C8—C9—H9B	108.9	C29—C28—H28A	108.4
C10—C9—H9B	108.9	C27—C28—H28A	108.4
H9A—C9—H9B	107.7	C29—C28—H28B	108.4
C11—C10—C9	114.1 (3)	C27—C28—H28B	108.4
C11—C10—H10A	108.7	H28A—C28—H28B	107.5
C9—C10—H10A	108.7	C28—C29—C30	114.0 (3)
C11—C10—H10B	108.7	C28—C29—H29A	108.8
C9—C10—H10B	108.7	C30—C29—H29A	108.8
H10A—C10—H10B	107.6	C28—C29—H29B	108.8
C12—C11—C10	114.1 (3)	C30—C29—H29B	108.8
C12—C11—H11A	108.7	H29A—C29—H29B	107.7
C10—C11—H11A	108.7	C31—C30—C29	114.7 (3)
C12—C11—H11B	108.7	C31—C30—H30A	108.6
C10—C11—H11B	108.7	C29—C30—H30A	108.6
H11A—C11—H11B	107.6	C31—C30—H30B	108.6
C11—C12—C13	115.1 (3)	C29—C30—H30B	108.6
C11—C12—H12A	108.5	H30A—C30—H30B	107.6
C13—C12—H12A	108.5	C30—C31—C32	114.0 (3)
C11—C12—H12B	108.5	C30—C31—H31A	108.7
C13—C12—H12B	108.5	C32—C31—H31A	108.7
H12A—C12—H12B	107.5	C30—C31—H31B	108.7
C14—C13—C12	115.4 (4)	C32—C31—H31B	108.7
C14—C13—H13A	108.4	H31A—C31—H31B	107.6
C12—C13—H13A	108.4	C33—C32—C31	115.1 (3)
C14—C13—H13B	108.4	C33—C32—H32A	108.5
C12—C13—H13B	108.4	C31—C32—H32A	108.5
H13A—C13—H13B	107.5	C33—C32—H32B	108.5
C13—C14—C15	114.9 (4)	C31—C32—H32B	108.5
C13—C14—H14A	108.5	H32A—C32—H32B	107.5
C15—C14—H14A	108.5	C32—C33—C34	114.7 (3)
C13—C14—H14B	108.5	C32—C33—H33A	108.6
C15—C14—H14B	108.5	C34—C33—H33A	108.6
H14A—C14—H14B	107.5	C32—C33—H33B	108.6
C14—C15—C16	115.5 (4)	C34—C33—H33B	108.6
C14—C15—H15A	108.4	H33A—C33—H33B	107.6
C16—C15—H15A	108.4	C35—C34—C33	115.2 (3)
C14—C15—H15B	108.4	C35—C34—H34A	108.5
C16—C15—H15B	108.4	C33—C34—H34A	108.5
H15A—C15—H15B	107.5	C35—C34—H34B	108.5
C17—C16—C15	116.2 (4)	C33—C34—H34B	108.5
C17—C16—H16A	108.2	H34A—C34—H34B	107.5
C15—C16—H16A	108.2	C34—C35—C36	114.7 (3)
C17—C16—H16B	108.2	C34—C35—H35A	108.6
C15—C16—H16B	108.2	C36—C35—H35A	108.6
H16A—C16—H16B	107.4	C34—C35—H35B	108.6
C16—C17—C18	116.1 (4)	C36—C35—H35B	108.6
C16—C17—H17A	108.3	H35A—C35—H35B	107.6
C18—C17—H17A	108.3	C35—C36—H36A	109.5
C16—C17—H17B	108.3	C35—C36—H36B	109.5
C18—C17—H17B	108.3	H36A—C36—H36B	109.5
H17A—C17—H17B	107.4	C35—C36—H36C	109.5
C17—C18—H18A	109.5	H36A—C36—H36C	109.5
C17—C18—H18B	109.5	H36B—C36—H36C	109.5
H18A—C18—H18B	109.5	O2—N1—O1	123.0 (3)
C17—C18—H18C	109.5	O2—N1—C1	119.6 (3)
H18A—C18—H18C	109.5	O1—N1—C1	117.4 (3)
H18B—C18—H18C	109.5	O5—N2—O4	122.5 (3)
C20—C19—C24	121.5 (3)	O5—N2—C19	119.4 (4)
C20—C19—N2	119.5 (4)	O4—N2—C19	118.1 (4)
C24—C19—N2	118.9 (4)	C4—O3—C7	118.9 (2)
C19—C20—C21	119.4 (3)	C22—O6—C25	118.2 (3)

### Hydrogen-bond geometry (Å, °)

**Table:**

*D*—H···*A*	*D*—H	H···*A*	*D*···*A*	*D*—H···*A*
C3—H3···O4^i^	0.93	2.69	3.329 (4)	127
C6—H6···O2^ii^	0.93	2.60	3.375 (4)	141
C20—H20···O5^iii^	0.93	2.52	3.372 (5)	152

Symmetry codes: (i) *x*−1, *y*, *z*; (ii) −*x*+3, −*y*, −*z*+2; (iii) −*x*+4, −*y*−1, −*z*+2.
